# Combining PIM‐2 and PARP1 Inhibitors Induces MICA Expression on Multiple Myeloma Cells to Activate NK Cells through NKG2D Binding

**DOI:** 10.1002/advs.202502448

**Published:** 2025-06-25

**Authors:** Zhaoyun Liu, Wenhui Lei, Xiaohan Liu, Hui Liu, Kai Ding, Jia Song, Rong Fu

**Affiliations:** ^1^ Department of Hematology Tianjin Medical University General Hospital 154 Anshan Street Tianjin 300052 P. R. China; ^2^ Department of Nephrology Fifth Affiliated Hospital of Wenzhou Medical University Lishui Zhejiang 323000 P. R. China; ^3^ Tianjin Key Laboratory of Bone Marrow Failure and Malignant Hemopoietic Clone Control Tianjin 300052 P. R. China; ^4^ Tianjin Institute of Hematology Tianjin 300052 P. R. China; ^5^ State Key Laboratory of Experimental Hematology Tianjin P. R. China

**Keywords:** multiple myeloma, NK Cells, PARP1 inhibitor, PIM‐2 inhibitor

## Abstract

While immunogenic death in tumor cells activates specific anti‐tumor T cells, the activation of non‐specific natural killer (NK) cells through tumor interactions remains underexplored. This study investigates how inducing DNA damage in multiple myeloma (MM) cells leads to MICA overexpression, facilitating NKG2D binding on NK cells.We analyzed the relationship between PIM‐2, PARP1, and MM prognosis using public data and clinical samples. An in vitro co‐culture of MM and NK cells assessed the effects of SMI‐16a (PIM‐2 inhibitor) and ABT888 (PARP1 inhibitor) on tumor proliferation and apoptosis, focusing on DNA damage, MICA expression, and NK cell functionality. An NSG mouse model evaluated the combined effects on tumor growth and NK cell activity.The combination significantly increased DNA damage, marked by elevated pH2AX, and enhanced NK cell functional markers, including perforin and granzyme B. Increased DNA damage correlated with heightened MICA expression, activating NK cells via the NKG2D/MICA signaling pathway. PIM‐2 and PARP1 inhibitors synergistically induce MICA expression on MM cells, enhancing NK cell activation through NKG2D binding, offering a promising therapeutic strategy for MM patients.

## Graphical abstract

The findings suggest that the combination of PIM‐2 and PARP1 inhibitors can induce MICA expression on MM cells, thereby activating NK cells through NKG2D binding. This process may restore NK cell function and serve as a potential therapeutic approach for MM patients.

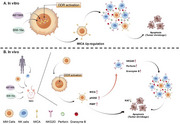

## 1 Introduction

Multiple myeloma (MM) is a hematologic malignancy characterized by the clonal proliferation of abnormal plasma cells, accounting for ≈1% of all global cancer cases and ≈10% of hematologic malignancies.^[^ ^1^, ^2^, ^3^, ^4^ ^]^ Significant advancements in MM treatment have been made in recent years due to progress in medical technology. Immunomodulatory drugs such as thalidomide and lenalidomide, proteasome inhibitors like bortezomib, anti‐CD38 monoclonal antibodies, and chimeric antigen receptor T (CAR‐T) cell therapies have notably improved patient outcomes.^[^ ^2^, ^5^, ^6^ ^]^ While modern therapies have significantly improved progression‐free survival rates, MM remains an incurable malignancy with inevitable relapse patterns. This underscores the critical need for novel therapeutic strategies capable of achieving durable complete remission and potentially transitioning toward functional cure paradigms, as recently conceptualized in the International Myeloma Working Group consensus.^[^ ^7^, ^8^ ^]^

The PIM kinase family, a key subgroup of serine/threonine kinases, was initially studied in association with lymphomas induced by the Moloney murine leukemia virus.^[^ ^9^ ^]^ Recent research has identified elevated expression levels of PIM‐2 in MM, which promotes the proliferation and survival of malignant plasma cells.^[^ ^10^, ^11^, ^12^ ^]^ Inhibitors targeting PIM‐2 have shown efficacy in reducing tumor cell growth and survival by specifically inhibiting PIM‐2 activity in MM cells, demonstrating substantial antitumor effects.^[^ ^11^, ^13^ ^]^ Consequently, PIM‐2 inhibitors represent a promising therapeutic avenue with significant potential for treating MM and associated bone diseases.^[^ ^11^, ^14^, ^15^ ^]^ Several PIM‐2 inhibitors, including NCT02078609, NCT02160951, and NCT02144038, are currently undergoing clinical trials to assess their effectiveness in managing multiple myeloma.^[^ ^16^ ^]^

Natural killer (NK) cells, classified as the first subtype of innate lymphoid cells (ILCs), exhibit a rapid and potent immune response against viral infections and malignant cells, including direct cytotoxicity targeting tumor cells.^[^ ^17^, ^18^ ^]^ Numerous studies have confirmed the significant antitumor activity of NK cells against MM cells.^[^ ^19^ ^]^ Enhancing the tumor‐killing capacity of NK cells has therefore become a central focus and challenge in cancer research. Certain studies have shown that specific drugs can upregulate the expression of NKG2D ligands, such as MICA/B and ULBP1‐3, in myeloma cells through DNA damage response pathways mediated by ATM and ATR. This upregulation activates NK cell cytotoxicity through the NKG2D/MICA signaling axis.^[^ ^20^ ^]^ Thus, activating the tumor‐killing function of NK cells through the NKG2D/MICA signaling axis is considered a promising approach to enhancing drug treatment efficacy.

This report introduces a novel therapeutic strategy combining the PIM‐2 inhibitor SMI‐16a and the PARP1 inhibitor ABT888. This combination induces DNA damage in MM cells and augments the tumor‐killing function of NK cells via the NKG2D/MICA signaling axis. The proposed approach significantly enhances the therapeutic efficacy of the drug combination and provides an experimental foundation for developing innovative treatments for multiple myeloma.

## 2 Results

### 2.1 Expression of PIM‐2 and PARP1 in MM Cells is Associated with the Prognosis of MM Patients

**Figure 1:**
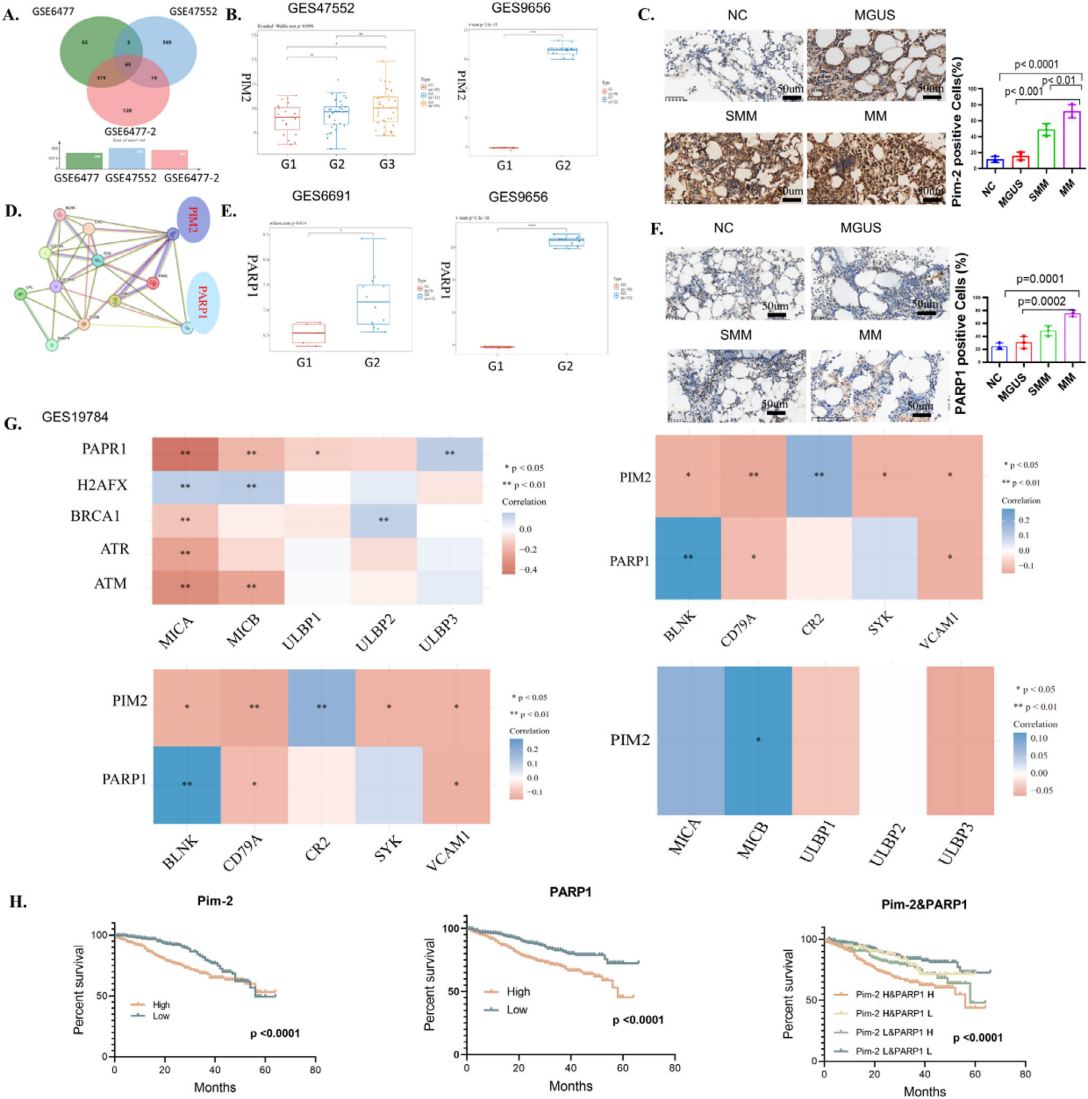
The expression levels of PIM‐2 and PARP1 in multiple myeloma (MM) are associated with the regulation of lymphocyte proliferation and apoptosis, and they correlate with prognostic outcomes in MM. A) Intersection analysis of differentially expressed genes from datasets GSE6477 and GSE47552 identified 65 upregulated genes in myeloma compared to normal bone marrow, including key genes involved in proliferation and apoptosis, RB1 and BNIP3. B) In the GSE47552 dataset, PIM‐2 expression is significantly higher in MM compared to monoclonal gammopathy of undetermined significance (MGUS) (G1: MGUS; G2: smoldering MM; G3: MM). Additionally, analysis of GSE9656 indicates that PIM‐2 expression is markedly increased in MM when compared to normal bone marrow (G1: normal bone marrow; G2: newly diagnosed MM). C) Immunohistochemical staining reveals a significant elevation in PIM‐2 expression in MM relative to normal bone marrow, MGUS, and smoldering MM tissues. D) Protein–protein interaction (PPI) analysis identifies functional interactions among PIM‐2, PARP1, and crucial proteins that are involved in lymphocyte proliferation and apoptosis pathways. E) Data from GSE6691 and GSE9656 demonstrate that PARP1 expression is significantly heightened in MM compared to normal bone marrow. F) Immunohistochemical analysis confirms that PARP1 levels are notably increased in MM compared to normal bone marrow, MGUS, and smoldering MM. G) Analysis of data from GSE19784 indicates that PIM‐2 and PARP1 are associated with apoptotic pathways. H) Insights from the Commpass database suggest that elevated PIM‐2 expression serves as a negative prognostic indicator, while high PARP1 expression functions as an independent prognostic factor for unfavorable clinical outcomes in MM patients. Importantly, patients exhibiting elevated levels of both PIM‐2 and PARP1 have the poorest prognoses.

Differential gene expression analysis was performed using datasets GSE6477 and GSE47552 from the GEO database to compare newly diagnosed multiple myeloma (MM), relapsed MM, and normal bone marrow plasma cells. A total of 65 commonly upregulated genes were identified, including key regulators of cell proliferation and apoptosis, such as RB1 and BNIP3 (**Figure** **1**A). Additional analysis of datasets GSE6691, GSE47552, and GSE9656, as well as single‐cell sequencing data GSE193531 (see Supporting Information), demonstrated that the expression levels of PIM‐2 (Figure 1B) and PARP1 (Figure 1E) were significantly elevated in multiple myeloma (MM) compared to normal bone marrow and monoclonal gammopathy of undetermined significance (MGUS), suggesting that both proteins are integral to the pathogenesis and progression of multiple myeloma.

In a retrospective analysis, formalin‐fixed paraffin‐embedded bone marrow tissues from patients diagnosed with MM, smoldering myeloma (SMM), and MGUS were examined. Three cases, each from normal control bone marrow (NC), MGUS, SMM, and MM, were selected, and immunohistochemical techniques were employed to evaluate the expression of PARP1 and PIM2. Results demonstrated a higher positive rate of PARP1 and PIM2 expression in MM compared to NC, MGUS, and SMM (Figure 1C/F).

To investigate the relationship between PIM‐2, PARP1, and previously identified upregulated genes, the STRING database was utilized to analyze functional interactions involving PIM‐2, PARP1, and proteins linked to lymphocyte proliferation (CD79A, CR2, VCAM1, SYK, BLNK) as well as key apoptotic pathway proteins (RB1, BNIP3). This analysis revealed significant functional interactions (PPI enrichment with a P‐value of 2.34e‐10) between PIM‐2, PARP1, and these critical proteins involved in lymphocyte proliferation and apoptosis pathways (Figure 1D). Additionally, analysis of the GSE19784 dataset confirmed the correlation of PIM‐2 and PARP1 with apoptotic pathways and lymphocyte proliferation (Figure 1G).

To assess the prognostic significance of PIM‐2 and PARP1 in multiple myeloma, gene expression and follow‐up data for 787 MM patients were accessed from the Commpass database. Median expression levels of PIM‐2 and PARP1 were used as cutoff values to categorize patients into high‐expression (H) and low‐expression (L) groups. Kaplan–Meier survival analysis indicated that high PIM‐2 expression is associated with poor prognosis (Chi‐square = 28.4, *p* < 0.001). Furthermore, high PARP1 expression was identified as an independent prognostic factor for unfavorable outcomes in MM patients (Chi‐square = 17.52, *p* < 0.001). Patients with elevated levels of both PARP1 and PIM‐2 exhibited the poorest prognosis (Chi‐square = 25.11, *p* < 0.001) (Figure 1H).

### 2.2 The Combination of PIM‐2 and PARP1 Inhibitors Significantly Inhibits Cell Viability and Promotes Apoptosis in Multiple Myeloma Cells

**Figure 2:**
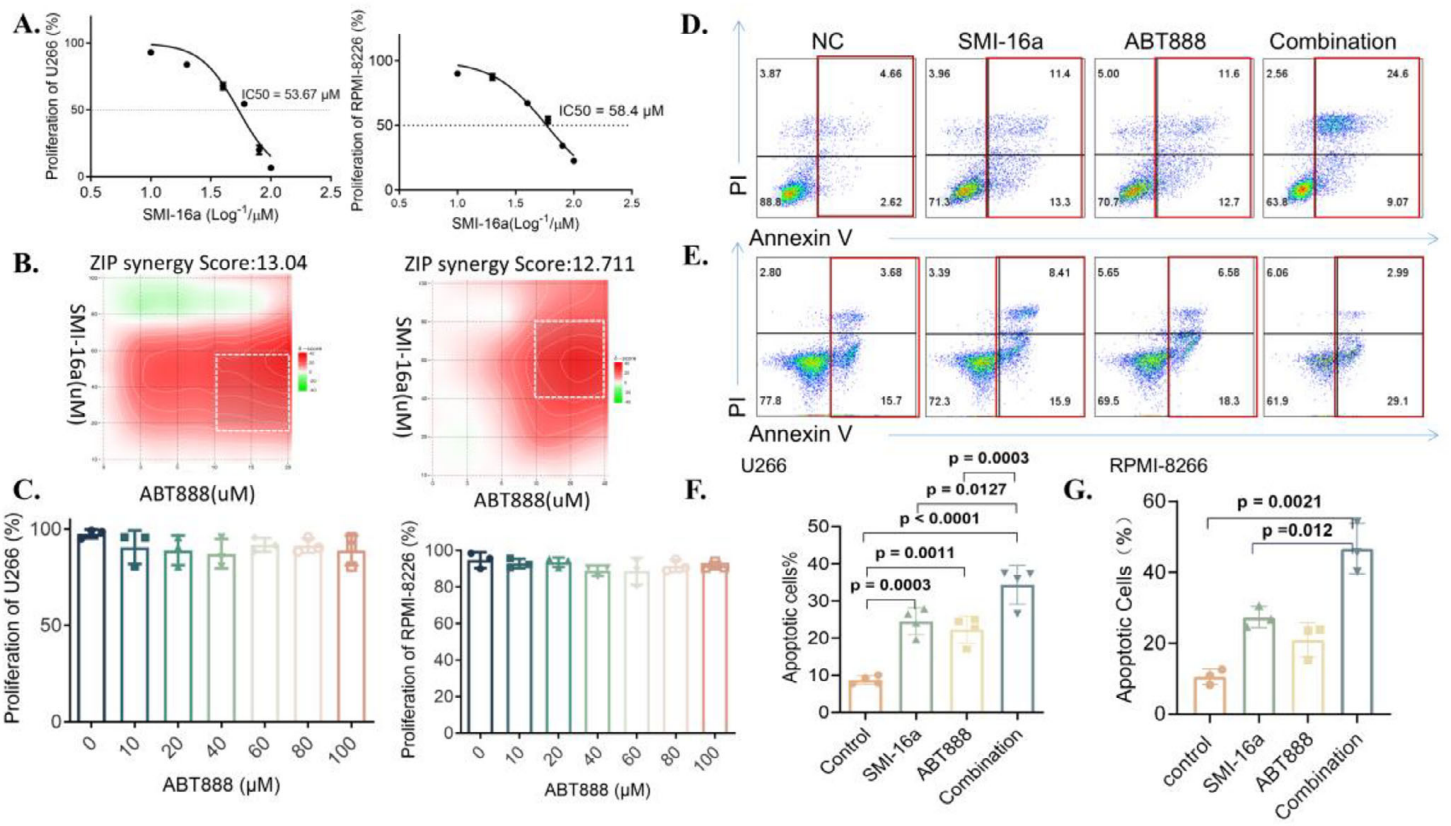
The combination of SMI‐16a and ABT888 markedly inhibits cell viability and induces apoptosis in MM cells compared to SMI‐16a treatment alone. A) IC50 values of SMI‐16a in U266 and RPMI‐8266 cell lines when administered as a monotherapy. B) Heatmap illustrating the response to drug combinations. SMI‐16a and ABT888 demonstrate a synergistic effect in RPMI‐8266 and U266 cells. The gradient of the red areas indicates the intensity of synergy. C) Monotherapy with ABT888 (Veliparib) does not significantly affect MM cell viability. D) Flow cytometry analysis illustrates the effect of the drug combination on apoptosis rates in U266 cells. E) Flow cytometry analysis of apoptosis rates in RPMI‐8266 cells following combined treatment. F) Comparative analysis of apoptosis rates in U266 cells. G) Comparative analysis of apoptosis rates in RPMI‐8266 cells.

To evaluate the inhibitory effects of the PIM‐2 inhibitor SMI‐16a and the PARP1 inhibitor ABT888 (Veliparib) on tumor cell viability, the CCK‐8 assay was conducted. The results indicated that the antiproliferative effect of SMI‐16a was concentration‐dependent, with greater inhibition observed at higher concentrations. Conversely, ABT888 monotherapy did not produce a significant reduction in MM cell proliferation (**Figure** **2**C). The ZIP synergy scores were calculated using SynergyFinder software. A score greater than 0 indicates synergy, while a score exceeding 10 is considered to reflect strong synergy. As shown in the figure below, the combined application of the two drugs yields a ZIP synergy score greater than 10, indicating a synergistic effect (Figure 2B).

To investigate whether the combination of SMI‐16a and ABT888 could improve apoptosis rates in MM cells compared to SMI‐16a monotherapy, four experimental groups were established: Control, ABT888 (40 µM), SMI‐16a (80 µM), and the Combination group. After 48 h of treatment, cells were harvested, and apoptosis rates were measured using an Annexin V‐PI apoptosis detection kit. The results demonstrated that combination therapy significantly increased the apoptosis rate in RPMI‐8266 and U266 cells compared to SMI‐16a monotherapy, underscoring the potential synergistic effect of the two inhibitors (Figure 2D‐G).

### 2.3 Synergistic Effects of PIM‐2 and PARP1 Inhibitors on DNA Damage and MICA Expression in Multiple Myeloma Cells

**Figure 3:**
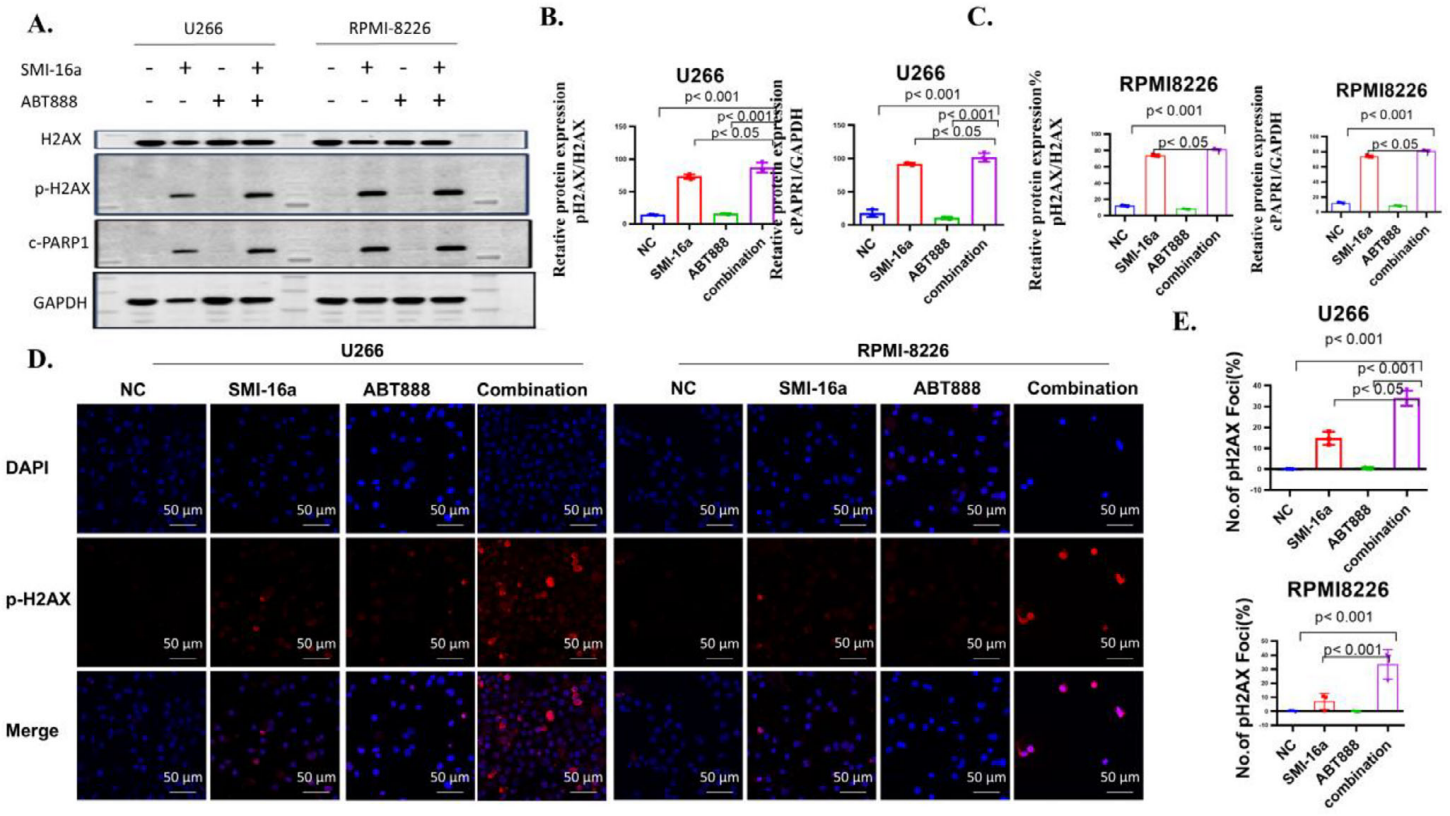
The combined treatment of SMI‐16a and ABT888 significantly boosts the expression of DNA damage markers p‐H2AX and PARP1 in MM cells compared to SMI‐16a alone. A) Western blot analysis reveals that the combination treatment notably elevates the expression levels of p‐H2AX and PARP1 compared to SMI‐16a monotherapy. B,C) Quantitative analysis of p‐H2AX and PARP1 expression levels from Western blot results. D) Immunofluorescence images illustrating p‐H2AX expression in U266 and RPMI‐8266 cells. E) Quantitative assessment of p‐H2AX expression in U266 and RPMI‐8266 cells via immunofluorescence.

To determine whether the combination of SMI‐16a and ABT888 enhances the expression of the DNA damage marker p‐H2AX and the DNA repair protein PARP1 compared to SMI‐16a monotherapy, Western blot analysis was performed. The results demonstrated that co‐treatment with both inhibitors significantly increased the expression levels of p‐H2AX and PARP1 compared to SMI‐16a alone (**Figure** **3**A‐C). Immunofluorescence assays conducted on RPMI‐8266 and U266 cells corroborated these findings, showing a significant elevation in p‐H2AX expression after 48 h of combination treatment (Figure 3D,E). These observations suggest a synergistic effect of the two inhibitors in inducing DNA damage.

**Figure 4:**
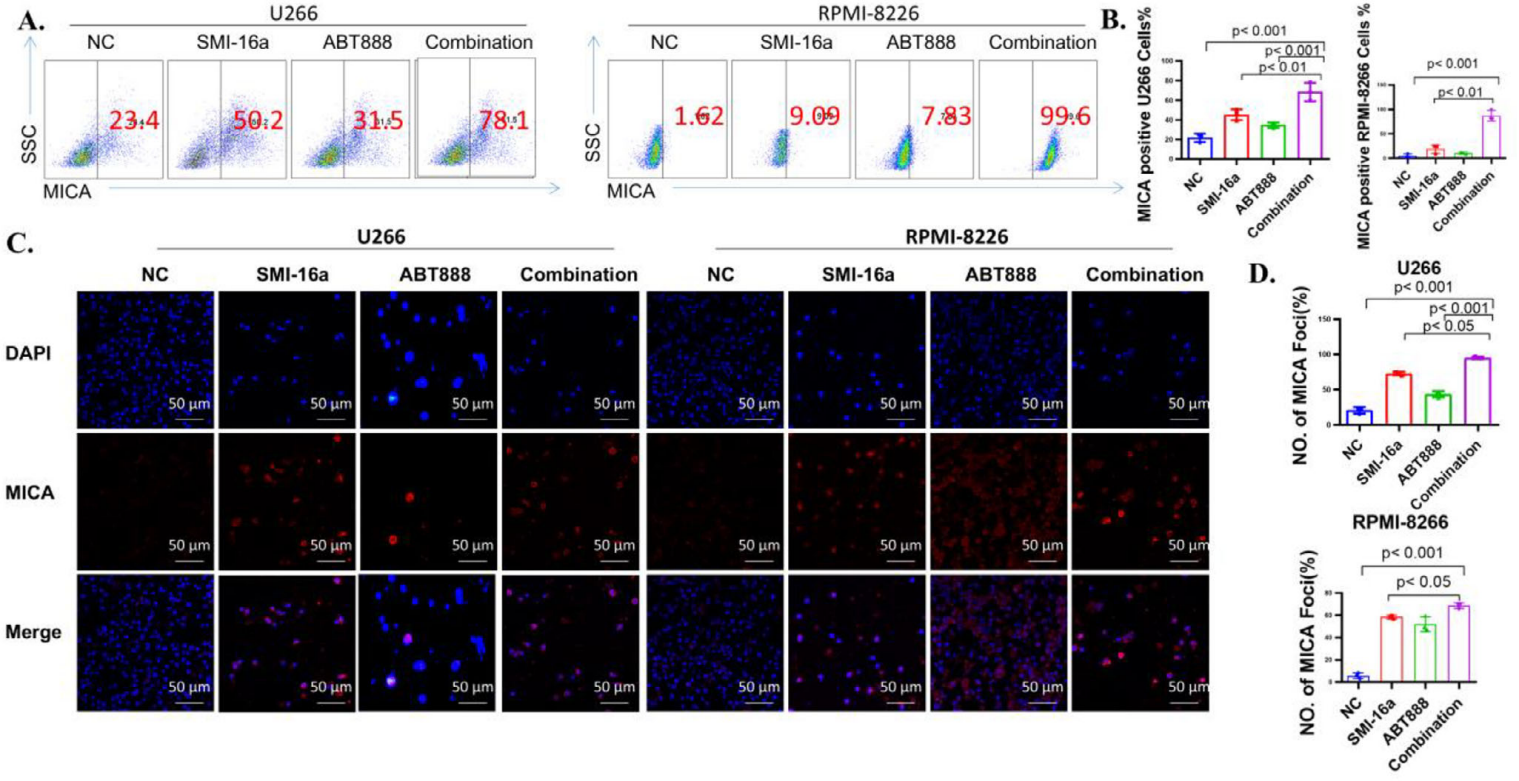
The combination of SMI‐16a and ABT888 enhances MICA expression more than SMI‐16a alone. A) Flow cytometry analysis of MICA expression in U266 and RPMI‐8266 cells. B) Comparative assessment of MICA expression levels in U266 and RPMI‐8266 cell lines. C) Immunofluorescence imaging of MICA expression in U266 and RPMI‐8266 cells. D) Comparative evaluation of MICA expression levels in U266 and RPMI‐8266 cells using immunofluorescence.

To evaluate whether the combination treatment enhances MICA expression compared to SMI‐16a monotherapy, RPMI‐8266 and U266 cells were treated with SMI‐16a (80 µM) and ABT888 (40 µM) either individually or in combination for 48 h. Flow cytometric analysis revealed a significant increase in MICA expression following the combined treatment compared to SMI‐16a alone (**Figure** **4**A,B). This result was further supported by immunofluorescence analysis, which confirmed a notable elevation in MICA expression after the 48‐h combination treatment relative to SMI‐16a monotherapy (Figure 4C,D), aligning with the flow cytometry findings.

### 2.4 The Combination of PIM‐2 and PARP1 Inhibitors Enhances NK Cell Function and Apoptosis of MM Cells in In Vitro Co‐Culture Systems

**Figure 5:**
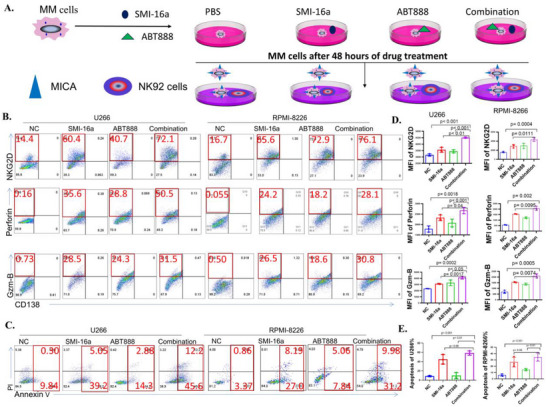
In vitro co‐culture experiments show that the combination of SMI‐16a and ABT888 markedly increases the expression of functional markers in NK‐92 cells, such as perforin, granzyme B, and NKG2D, compared to SMI‐16a alone, while also inducing apoptosis in multiple myeloma (MM) cells. A) Overview of the experimental workflow. B) Flow cytometric analysis of perforin, granzyme B, and NKG2D expression in NK‐92 cells. C) Flow cytometric evaluation of apoptosis in U266 and RPMI‐8266 cells within the co‐culture system. D) Quantitative comparison of perforin, granzyme B, and NKG2D expression in U266 and RPMI‐8266 cells. E) Comparative assessment of apoptosis rates in U266 and RPMI‐8266 cells.

**Figure 6:**
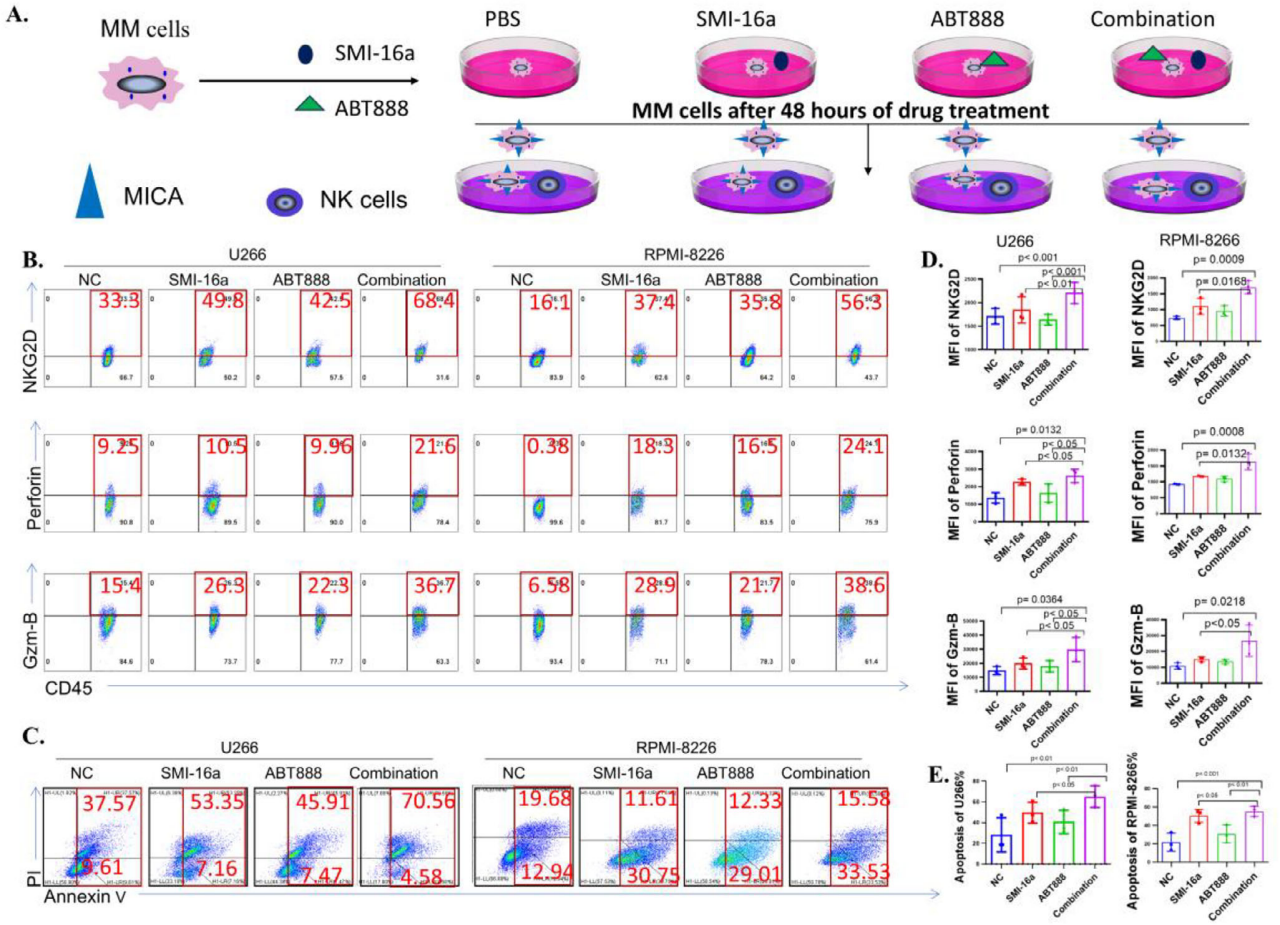
In vitro co‐culture experiments reveal that the combination of SMI‐16a and ABT888 significantly boosts the expression of functional markers, such as perforin, granzyme B, and NKG2D, in natural killer (NK) cells compared to treatment with SMI‐16a alone. Furthermore, this combination induces apoptosis in multiple myeloma (MM) cells. A) Overview of the experimental workflow. B) Flow cytometric analysis quantifying perforin, granzyme B, and NKG2D expression in NK cells. C) Flow cytometric evaluation of apoptosis in U266 and RPMI‐8266 cells within the co‐culture system. D) Quantitative comparison of perforin, granzyme B, and NKG2D expression levels in U266 and RPMI‐8266 cells. E) Comparative analysis of apoptosis rates in U266 and RPMI‐8266 cells.

To examine whether the combination of SMI‐16a and ABT888 enhances NK cell function more effectively than SMI‐16a alone, a co‐culture system was established using NK‐92 or NK cells with RPMI‐8266 and U266 cells at a 1:2 ratio. Cells were divided into four treatment groups: Control, ABT888 (40 µM), SMI‐16a (80 µM), and the Combination group. After 48 h of treatment, NK cell functionality was assessed via flow cytometry. Bone marrow myeloma cells were identified using PE‐CD138, while NK/NK‐92 cells were stained with antibodies targeting Perforin (APC anti‐human), Granzyme B (FITC anti‐human/mouse), and NKG2D (Percp Cy5.5). The results indicated that the Combination group exhibited significantly higher levels of functional markers Perforin, Granzyme B, and NKG2D in NK‐92 and NK cells compared to monotherapy groups with ABT888 or SMI‐16a (**Figures** **5**B and **6**B).

To further validate whether the combination of SMI‐16a and ABT888 enhances NK cell functionality and promotes apoptosis in multiple myeloma cells, a co‐culture system was again utilized with RPMI‐8266 and U266 cells alongside NK‐92 or NK cells. The cells were divided into the same treatment groups, and after 48 h, APC‐CD45 staining was employed to identify NK/NK‐92 cells, accounting for the reduction in CD138 expression associated with myeloma cell apoptosis. By excluding CD45‐negative myeloma cells, apoptosis was measured using the Annexin V‐PI apoptosis detection kit. Statistical analysis via a two‐sample t‐test revealed a significant increase in apoptosis rates in the Combination group compared to the ABT888 and SMI‐16a monotherapy groups (Figures 5C/E and 6C/E).

To comprehensively elucidate the molecular mechanisms underlying enhanced DNA damage‐induced apoptosis in multiple myeloma (MM) cells through concurrent inhibition of PIM‐2 and PARP1, we performed RNA sequencing on U266 and RPMI‐8226 cell lines following combined treatment with SMI‐16a and ABT888. Gene Ontology (GO) pathway enrichment analysis was conducted on differentially expressed genes (see Supporting Information), and key pathway‐associated proteins were subsequently validated via Western blot analysis (see Supporting Information). Our findings suggest that dual inhibition of PIM‐2 and PARP1 leads to elevated phosphorylation levels of ATM and ATR kinases, which in turn induces aberrant expression of downstream messenger RNAs (mRNAs). This dysregulation triggers DNA damage while simultaneously impairing DNA repair mechanisms, ultimately potentiating apoptotic cell death in MM cells.

### 2.5 In Vivo Experiments Demonstrate that the Combination of PIM‐2 and PARP1 Inhibitors Activates NK Cell Cytotoxicity via the NKG2D/MICA Signaling Axis

To investigate the combined effects of a PIM‐2 inhibitor and a PARP1 inhibitor on apoptosis and tumor growth in multiple myeloma (MM) cells in vivo, NSG mice were subcutaneously injected with 1 × 10^7^ RPMI‐8226 cells to establish a myeloma model. Tumor‐bearing mice were randomly assigned to six groups, each with four mice: Control, ABT888 monotherapy, SMI‐16a monotherapy, combination treatment (ABT888 and SMI‐16a), NK cell treatment, and combination + NK cell treatment.

**Figure 7:**
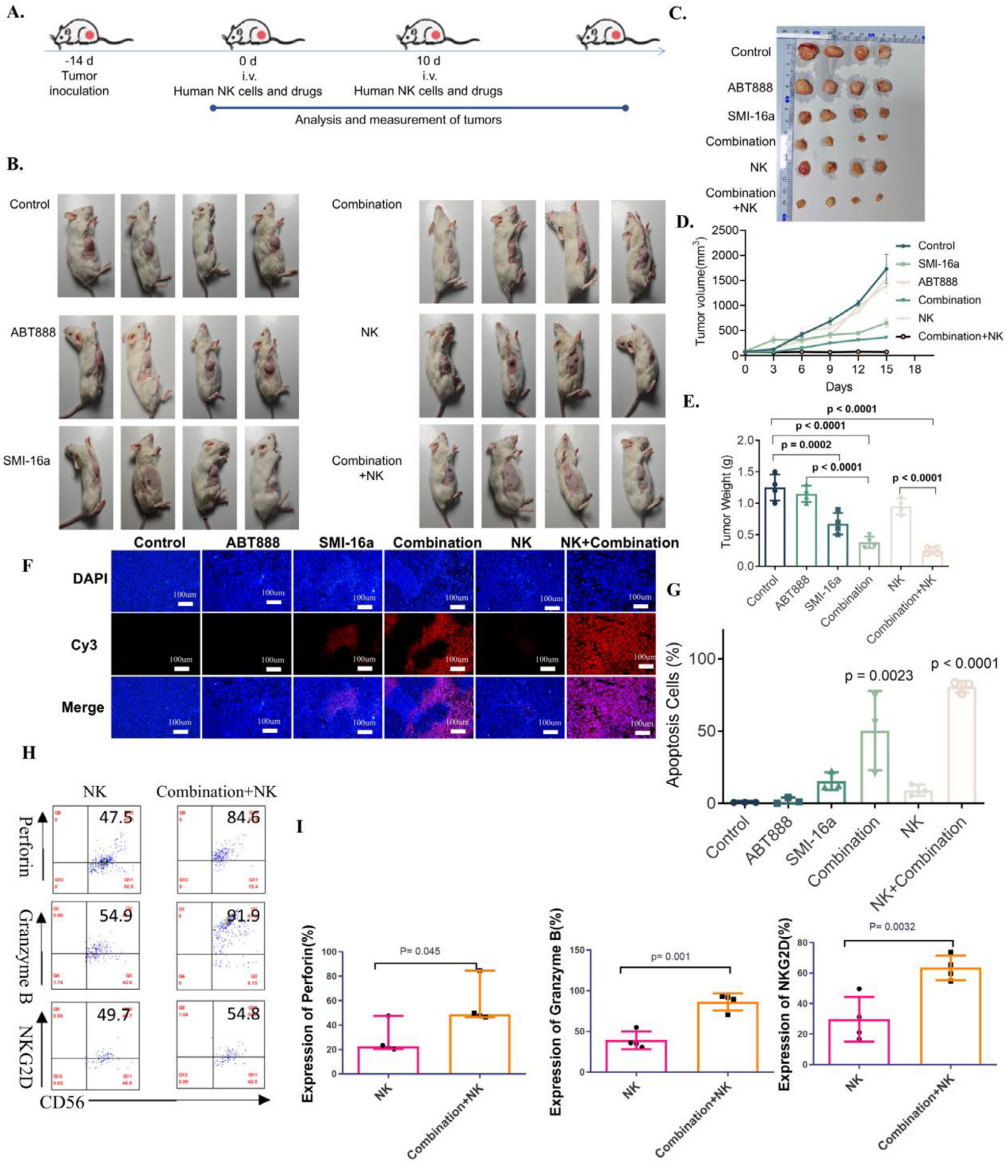
Animal studies show that the combination of SMI‐16a, ABT888, and NK cells significantly reduces tumor growth, promotes apoptosis in multiple myeloma (MM) cells, and increases the expression of functional markers in NK cells, such as perforin, granzyme B, and NKG2D, compared to SMI‐16a alone. A) Flowchart outlining the experimental protocol for the animal study. B) Images of subcutaneous tumor size after 12 days of treatment. C) Photographs comparing tumor size after subcutaneous dissection at 16 days of treatment. D) Graph showing changes in subcutaneous tumor volume over time. E) Comparison of tumor weight following subcutaneous dissection at 16 days of treatment. F) TUNEL assay highlighting apoptotic cells within tumor samples. G) Comparison of apoptosis rates among different treatment groups. H) Flow cytometric analysis of perforin, granzyme B, and NKG2D expression in NK cells. I) Quantitative comparison of perforin, granzyme B, and NKG2D expression levels in NK cells.

Tumor sizes were measured triweekly, and tumors were excised, photographed, and weighed post‐euthanasia. The results showed that ABT888 alone had minimal impact on tumor growth, while SMI‐16a monotherapy demonstrated moderate inhibition. The combination treatment significantly suppressed tumor growth, with the combination + NK cell group exhibiting the most substantial tumor inhibition (**Figure** **7**B‐E).

To evaluate in vivo apoptosis and NK cell‐mediated cytotoxicity, a one‐step TUNEL assay was conducted on paraffin‐embedded tumor sections. The results revealed a significant increase in tumor cell apoptosis in both the combination treatment and combination + NK cell groups, with the latter showing the most pronounced effect (Figure 7F,G).

Human NK cell functionality was further assessed by analyzing CD3‐CD56+ NK cells labeled with CD3 and CD56. Comparisons between the NK cell group and combination + NK cell group demonstrated a significant enhancement in NK cell activity in the latter. Granzyme B positivity rates increased from 35.88 ± 4.79% to 86.20 ± 10.57%, NKG2D from 26.38 ± 9.10% to 61.28 ± 11.58%, and Perforin from 21.83 ± 1.25% to 49.60 ± 3.73%, suggesting that the combination treatment robustly enhances NK cell‐mediated cytotoxicity (Figure 7H,I).

### 2.6 In Vivo Tumor Immunohistochemistry Detects Ki67, PARP1, p‐H2AX, MICA, and HE Staining on MM Cells

**Figure 8:**
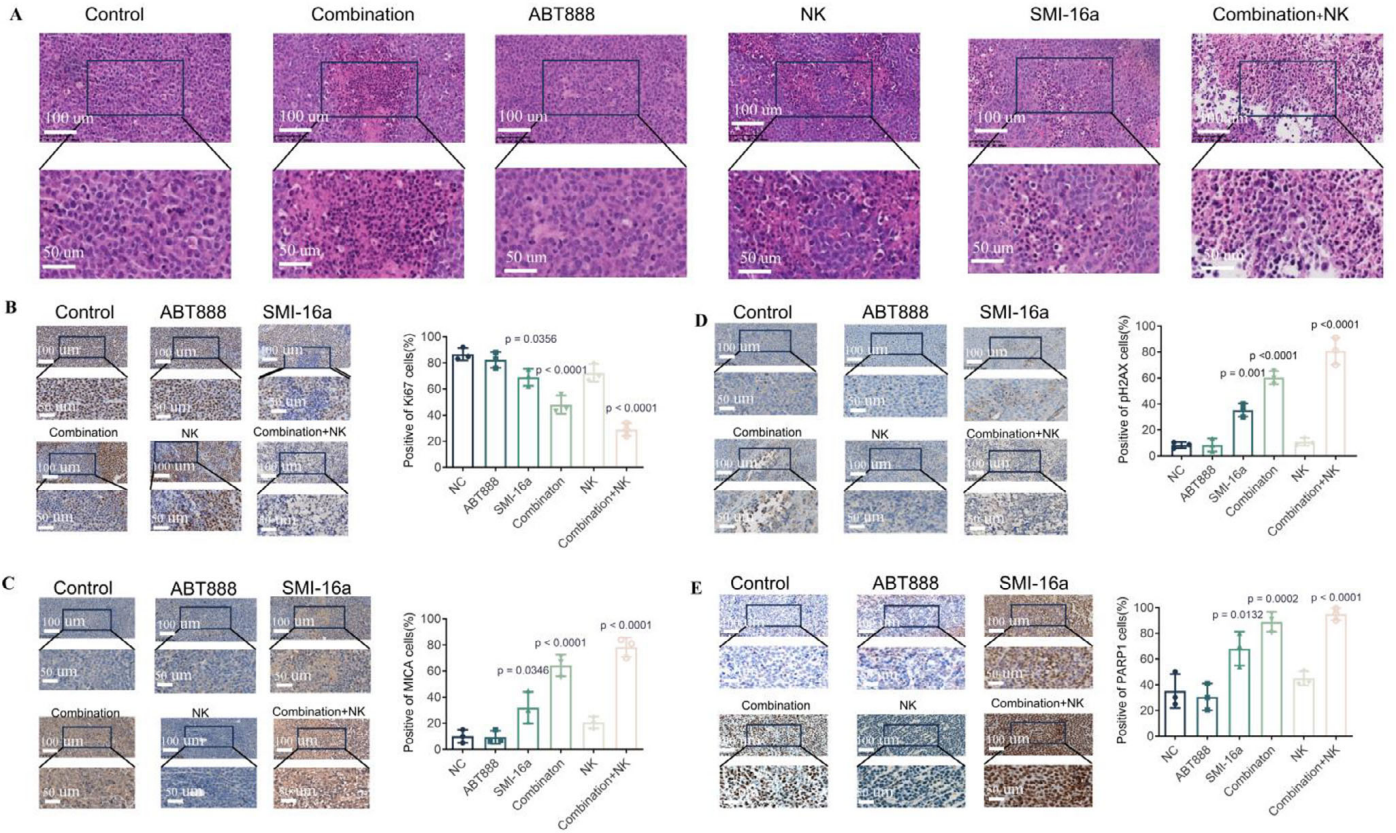
Hematoxylin and eosin (HE) staining reveals apoptotic morphological features in MM cells, while immunohistochemical analyses show the effects of combining SMI‐16a and ABT888 on the expression levels of Ki67, PARP1, p‐H2AX, and MICA. A) HE staining displays nuclear condensation and fragmentation in apoptotic cells within the combination + NK cell treatment group. B) Immunohistochemical staining for Ki67. C) Immunohistochemical staining for MICA. D) Immunohistochemical staining for p‐H2AX. E) Immunohistochemical staining for PARP1.

Immunohistochemical analysis was carried out in vivo to investigate the effects of the combined PIM‐2 inhibitor SMI‐16a and PARP1 inhibitor ABT888 on multiple myeloma (MM). The findings showed that the combination treatment notably increased the expression of DNA damage markers p‐H2AX and MICA when compared to SMI‐16a monotherapy. Additionally, PARP1 expression was found to be elevated, while Ki67, a marker of proliferation, exhibited decreased levels (**Figure** **8**B‐E). Hematoxylin and eosin (HE) staining highlighted morphological characteristics indicative of apoptosis, such as chromatin condensation and nuclear fragmentation (Figure 8A).

### 2.7 In Vivo Effects of PIM‐2 Combined with PARP1 Inhibitor on Liver and Kidney of Mice

To assess the potential hepatotoxicity and nephrotoxicity of the combined PIM‐2 inhibitor and PARP1 inhibitor, an experiment was conducted in which animals were euthanized for blood sample collection to evaluate liver and kidney function. Additionally, liver and kidney tissues were obtained for hematoxylin and eosin (HE) staining to identify any pathological alterations in hepatocytes and renal tissues.

**Figure 9:**
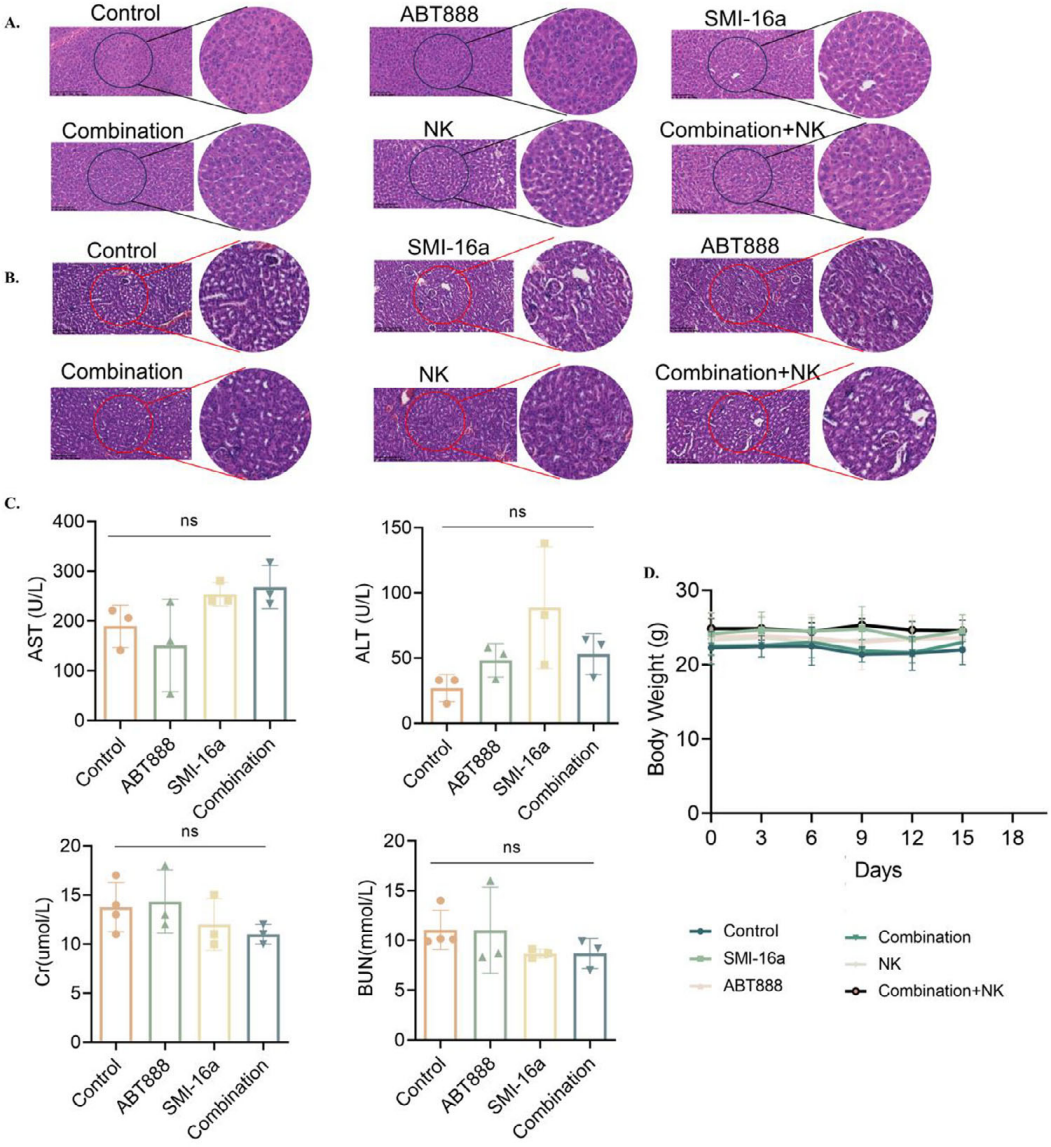
The combination of SMI‐16a and ABT888 does not adversely affect body weight, histopathological parameters of the liver and kidneys, or serum liver and kidney function indicators in the animal model.A:HE staining images of liver tissue.B:HE staining images of kidney tissue. C: Comparisons of serum ALT, AST, BUN, and creatinine levels among treatment groups.D:Impact of drug treatment on animal body weight.

The results revealed no statistically significant differences in liver and kidney function between the combination treatment group and the control group, as indicated by both the pathological findings and blood biochemical parameters (**Figure** **9**). These observations suggest that the combination of SMI‐16a and ABT888 does not have a detrimental effect on liver and kidney function compared to the control group.

## 3 Discussion

The PIM‐2 inhibitor has emerged as a promising therapeutic approach for multiple myeloma, as highlighted by extensive reports and ongoing clinical studies supporting its potential efficacy. Similarly, PARP1 inhibitors have shown significant antitumor activity in various cancers, including breast and ovarian cancers, primarily by disrupting DNA repair mechanisms.^[^ ^21^ ^]^ However, there is a noticeable gap in the literature regarding whether the combination of PIM‐2 inhibitors and PARP1 inhibitors could intensify DNA damage, enhance MICA expression on tumor cells, and subsequently activate NK cell‐mediated cytotoxicity. The findings presented demonstrate that the combined use of the PIM‐2 inhibitor SMI‐16a and the PARP1 inhibitor ABT888 leads to greater DNA damage compared to SMI‐16a alone, thereby facilitating NK cell cytotoxicity through the NKG2D/MICA signaling axis and inducing apoptosis in multiple myeloma cells. These results suggest that this combination may offer a beneficial therapeutic strategy for myeloma treatment.

Natural Killer (NK) cells are integral to the immune system, with their activation tightly regulated by a balance between inhibitory and activating receptors.^[^ ^22^, ^23^ ^]^ Among these receptors, NKG2D functions as a potent activator and is consistently expressed on all NK cells, as well as on NKT cells and certain T cell subsets. NKG2D uniquely recognizes stress‐induced self‐protein ligands, a mechanism termed “inducible self‐recognition”.^[^ ^24^ ^]^ In humans, these ligands primarily include MHC class I‐related proteins MICA and MICB, alongside ULBP family members. Notably, MICA, a critical NKG2D ligand, is significantly upregulated in response to cellular stressors such as DNA damage, viral infections, or malignancies.^[^ ^25^, ^26^ ^]^ This characteristic establishes the NKG2D/MICA signaling axis as a key mechanism enabling NK cells to detect and eliminate abnormal cells.^[^ ^27^ ^]^ Activation of this axis delivers strong activation signals, triggering NK cell‐mediated cytotoxicity against tumor cells.^[^ ^28^ ^]^ Moreover, studies have shown that the FDA‐approved histone deacetylase (HDAC) inhibitor panobinostat synergizes with MICA/B antibodies to enhance MICA/B surface expression on tumor cells, thereby improving NK cell cytotoxicity.^[^ ^29^ ^]^ These findings underscore the importance of exploring additional strategies to further augment MICA expression.

Recent literature underscores the critical role of the DNA damage response (DDR) in regulating the expression of NKG2D ligands, including MICA.^[^ ^30^ ^]^ While MICA is generally expressed at low levels in normal cells, its expression is significantly elevated in tumor cells experiencing DDR or persistent DDR signaling, thereby activating NK cells. In this context, engineered herpes simplex virus type 1 (HSV‐1) has been utilized to enhance DDR in glioblastoma (GBM) and glioblastoma stem‐like cells (GSC), demonstrating improved therapeutic outcomes.^[^ ^31^ ^]^ Inducing DNA damage in tumor cells has proven effective in promoting MICA expression, which subsequently enhances NK cell recognition and cytotoxicity against these cells. This approach has been validated in various experimental models, where DNA damage inducers led to marked upregulation of MICA and improved NK cell‐mediated tumor cell elimination.^[^ ^32^ ^]^ Chemotherapeutic agents further contribute to this process by inducing DNA damage and cellular senescence, indirectly enhancing NKG2D ligand expression and improving NK cell efficacy against tumors.^[^ ^33^ ^]^ The combination of DNA damage inducers with NK cell immunotherapy, therefore, holds potential for innovative tumor treatment strategies with enhanced therapeutic outcomes.

Experimental results corroborate these findings, as demonstrated through immunofluorescence and Western blot analysis, where the combination of SMI‐16a and ABT888 was shown to upregulate the DNA damage marker p‐H2AX alongside increased MICA expression, implicating the DNA damage pathway in MICA elevation.

PIM‐2 plays a pivotal role in DDR regulation, significantly influencing the survival and proliferation of multiple myeloma (MM) cells. Inhibitors of PIM‐2 have been shown to impact MM cell survival through the DNA damage pathway.^[^ ^30^, ^34^ ^]^ Further, the PIM inhibitor AZD1208, in combination with Akt inhibitors, exhibits synergistic antitumor effects in gastric cancer cell lines, potentially mediated via modulation of DNA damage repair pathways.^[^ ^35^ ^]^ These observations suggest that combining inhibitors of DNA damage repair enzymes may enhance the effectiveness of antimyeloma therapies.

Poly (ADP‐ribose) polymerase 1 (PARP1) inhibitors are an important class of targeted therapies that focus on the DNA damage response (DDR). Their antitumor effects primarily depend on inducing synthetic lethality in specific tumor types, especially those with defects in homologous recombination (HR) repair pathways, such as tumors containing mutations in BRCA1 or BRCA2 genes.^[^ ^21^, ^36^ ^]^ PARP1 inhibitors are widely used in the treatment of HR‐deficient tumors, including ovarian cancer, breast cancer—particularly triple‐negative breast cancer—and prostate cancer. Key inhibitors, including Olaparib, Niraparib, Rucaparib, and Talazoparib, have shown significant antitumor activity in clinical trials and have been approved by the U.S. Food and Drug Administration (FDA).

Despite the widespread use of PARP1 inhibitors across various cancers, the combined effects of PARP1 inhibitors and PIM‐2 inhibitors in enhancing DNA damage have not been fully explored. This study demonstrated that the simultaneous administration of SMI‐16a and ABT888 resulted in increased levels of the DNA damage marker p‐H2AX and PARP1 protein, compared to SMI‐16a alone, thereby enhancing MICA expression. Additionally, our co‐culture system of multiple myeloma (MM) cells and NK cells exhibited increased NK cell activation and higher apoptosis rates in MM cells, with in vivo experiments validating the in vitro results. Ultimately, in vivo studies confirmed that the combination of SMI‐16a and ABT888 with NK cells significantly reduced tumor growth.

Regarding drug safety, no significant differences in liver and kidney function were observed between the treatment and control groups. However, further clinical safety evaluations of SMI‐16a are recommended. Histopathological analysis of liver and kidney tissues did not show any evidence of toxicity linked to the drug.

While the experimental data indicate that the combination of SMI‐16a and ABT888 enhances NK cell activation through the NKG2D/MICA signaling axis by promoting DNA damage, several limitations of this study warrant consideration. First, the precise mechanisms by which PIM‐2 inhibitors induce DNA damage in multiple myeloma (MM) cells remain undefined. Second, the pathways that mediate the increased expression of MICA in response to DNA damage in MM cells are not fully elucidated. Finally, given that this is a preclinical study, the clinical relevance of these findings remains uncertain, underscoring the need for further investigations to validate these results.

We acknowledge that our findings, while promising, are positioned within a preclinical framework. Additionally, we emphasize the potential challenges, such as tumor resistance, that may necessitate the further development of supplementary therapeutic agents for clinical application.

In conclusion, this study demonstrates that the combination of SMI‐16a and ABT888 enhances NK cell‐mediated tumor killing through the NKG2D/MICA signaling axis, promoting DNA damage and resulting in a stronger antitumor response against multiple myeloma.

## 4 Conclusion

**Figure 10:**
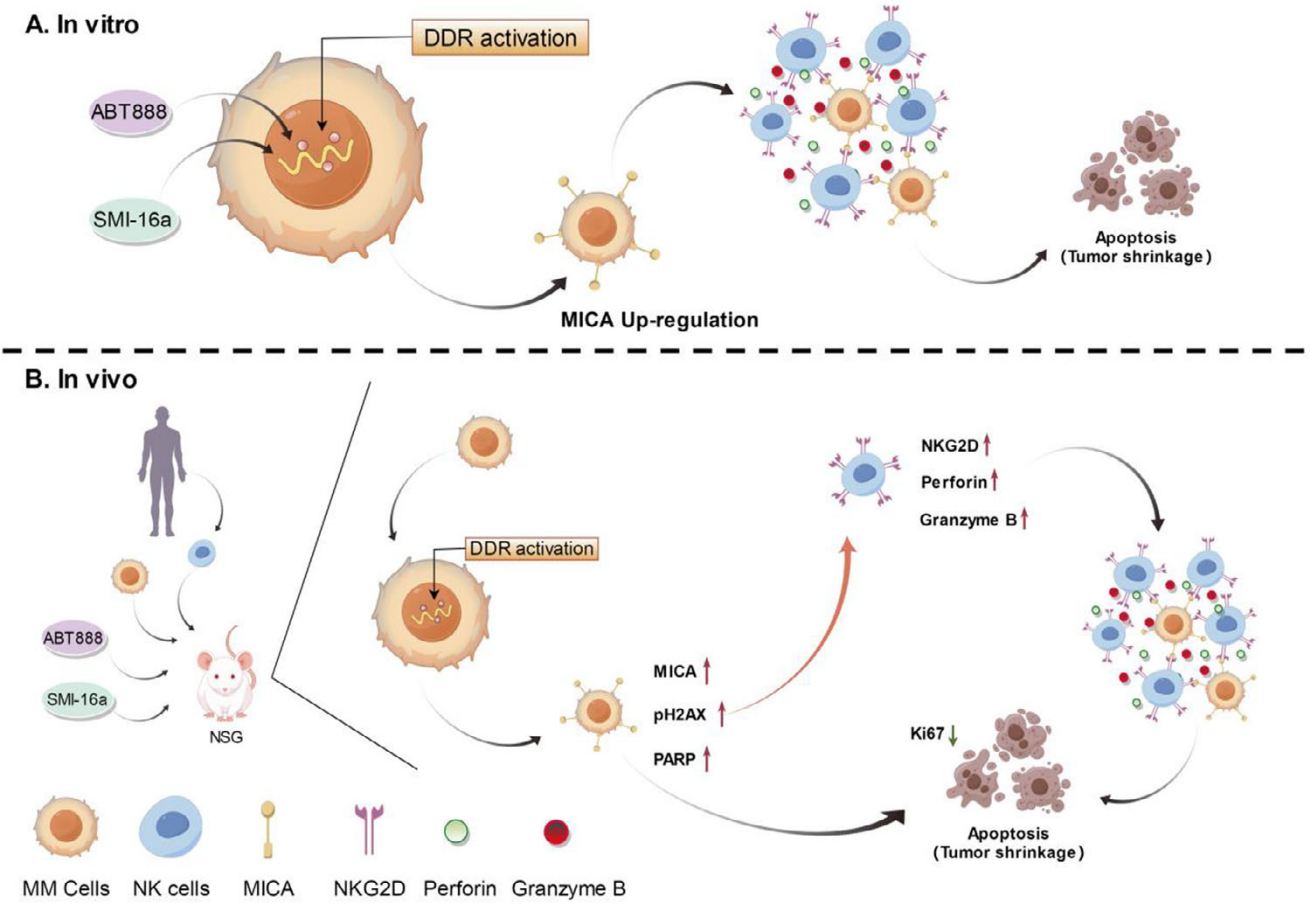
Graphical Abstract: Mechanistic illustration of the promotion of DNA damage in multiple myeloma (MM) cells by the combination of SMI‐16a and ABT888, alongside the activation of NK cell anti‐tumor capabilities through the NKG2D/MICA signaling axis. A) In vitro experiments demonstrated that the combination of SMI‐16a and ABT888 enhances the DNA damage response in MM cells, leading to increased expression of MICA on MM cells. This, in turn, activates NK cells by enhancing the secretion of perforin and Granzyme B, ultimately resulting in increased apoptosis of MM cells. B) In vivo experiments revealed that the administration of SMI‐16a and ABT888, combined with human‐derived NK cell injections into NSG mice with MM tumor models, induced DNA damage responses and elevated MICA expression in MM tumors. This effect similarly activated NK cell secretion of perforin and Granzyme B via the NKG2D/MICA signaling axis, thereby enhancing the anti‐tumor capacity of NK cells and reducing MM tumor volume.DDR: DNA damage response.(Illustrated by Figdraw).

The combination of the PIM‐2 inhibitor SMI‐16a and the PARP1 inhibitor ABT888 shows a synergistic effect in inducing DNA damage in MM cells, which results in increased MICA expression. Activation of the NKG2D/MICA signaling axis subsequently enhances NK cells expression of perforin, granzyme B, and NKG2D, improving their ability to kill tumor cells. Our findings suggest that the pairing of SMI‐16a and ABT888 enhances the DNA damage response, thereby boosting NK cell‐mediated antimyeloma activity through the NKG2D/MICA signaling axis (**Figure** **10**). Further studies are required to confirm these results and to better understand the mechanisms by which SMI‐16a and ABT888 induce DNA damage and subsequently elevate MICA expression in MM cells.

## 5 Experimental Section

### Analysis of Public Database Data

Gene expression profile data related to Multiple Myeloma (MM) and associated disorders were analyzed in this study, sourced from the Gene Expression Omnibus (GEO) database (https://www.ncbi.nlm.nih.gov/geo/), with data obtained in MINiML format. The datasets analyzed include GSE9656, GSE47552, GSE66691, and GSE19784. Clinical prognostic data for MM were collected from the COMMPASS database.

Gene correlation plots for gene pairs were generated using the R package ggstatsplot, while multi‐gene correlation plots were visualized using the pheatmap R package. A p‐value of less than 0.05 was considered statistically significant. Correlation coefficients ranged from −1 to 1, where negative values indicated an inverse relationship between gene expressions and positive values indicated a direct relationship. Additionally, the interactions between downstream proteins of these genes were explored using the STRING protein‐protein interaction database (https://cn.string‐db.org/).

Box plots were created using the boxplot function in the R environment to display data distribution and variability. Kaplan–Meier (KM) curves were applied to assess the statistical significance of survival differences between groups, with comparisons performed using the log‐rank test.

### Cell Culture

The RPMI‐8226 multiple myeloma cell line was acquired from the Chinese Academy of Sciences Cell Bank, while the U266 and NK‐92 cell lines were obtained from Wuhan Puno Life Technology Co., Ltd. U266 cells were grown in RPMI‐1640 medium containing 100 units mL^−1^ of penicillin, 100 µg mL^−1^ of streptomycin, and 10% fetal bovine serum. RPMI‐8226 cells were cultured in a medium supplemented with 20% Iscove's Modified Dulbecco's Medium (IMDM), under conditions similar to those used for U266 cells. The media were refreshed and cells passaged every 48 h, with a split ratio maintained between 1:2 and 1:3. NK‐92 cells were cultured in a specialized NK‐92 medium, strictly following the operational guidelines provided by Wuhan Puno Life Technology Co., Ltd. Primary human NK cells from the peripheral blood of MM patients were expanded ex vivo using feeder‐free cytokine cocktails (e.g., IL‐15 (50 ng mL^−1^) + IL‐21 (20 ng mL^−1^) + ALT‐803 (IL‐15 superagonist, 10 nM)), achieving greater than 40‐fold proliferation over 14 days while maintaining a purity of CD56+CD3‐ cells exceeding 90%.The PIM‐2 inhibitor SMI‐16a and the PARP1 inhibitor ABT‐888 (Veliparib) were obtained from MedChemExpress (https://www.medchemexpress.cn).

### Determination of IC50 Values of SMI‐16a and ABT‐888 on RPMI‐8226 and U266 Cells Using the CCK‐8 Assay

To evaluate the half‐maximal inhibitory concentration (IC50) of SMI‐16a and ABT‐888 on RPMI‐8226 and U266 cell lines, a Cell Counting Kit‐8 (CCK‐8) assay was carried out. RPMI‐8226 and U266 cells in the logarithmic growth phase were seeded at 3 × 10^5^ cells per well in 96‐well plates to maintain consistent experimental conditions. For each cell line, a range of concentrations for SMI‐16a and ABT‐888 was prepared, with three replicates for each treatment group. All treatment groups, including an untreated control group, were incubated for 48 h under standard conditions (37 °C, 95% air, and 5% carbon dioxide) to allow sufficient drug action on the target cells.

After the incubation, 10 µL of CCK‐8 reagent was added to each well in the dark, followed by a further 2 h of incubation under the same conditions. This allowed the CCK‐8 reagent to interact with the dehydrogenase enzymes of viable cells, generating a colorimetric product. The optical density (OD) of each well was measured at 450 nm using a microplate reader. The cell inhibition rates were calculated using the following formula:

(1) $$\begin{array}{ccc} \mathrm{Cell}\mathrm{Inhibition}\;\mathrm{Rate} & = & \left[\left(OD_{\mathrm{Experimental}}-OD_{\mathrm{Blank}}\right)/\left(OD_{\mathrm{Control}}-OD_{\mathrm{Blank}}\right)\right]\\ & & \times 100\% \end{array}$$

This method enabled the quantification of the inhibitory effects of different drug concentrations on cell growth. The inhibition rate data were analyzed using nonlinear regression in GraphPad Prism 8 software (GraphPad Software, Inc., San Diego, USA), facilitating the accurate determination of the IC50 values for both SMI‐16a and ABT‐888 in RPMI‐8226 and U266 cells, and providing a comprehensive assessment of the in vitro antitumor activity of these compounds.

### Immunohistochemistry, Immunofluorescence, and Flow Cytometry

Immunohistochemistry and immunofluorescence antibodies were sourced from Cell Signaling Technology (CST, USA), and antibodies for flow cytometry were obtained from BioLegend (USA). Functional markers of natural killer (NK) cells, including Granzyme B, Perforin, and NKG2D, were assessed using established protocols from the literature.^[^ ^37^ ^]^ Both immunohistochemical and immunofluorescence assays were performed according to referenced protocols.^[^ ^38^ ^]^ All fluorescence intensity measurements were performed using ImageJ software with rigorous standardization.This study was approved by the Medical Ethics Committee of Tianjin Medical University General Hospital (IRB2021‐KY‐279). Written informed consent was obtained from all participants prior to their inclusion in the study.

### Western Blotting

Protein expression analysis was conducted via Western blotting, following previously published protocols.^[^ ^39^ ^]^ A chemiluminescent detection kit was prepared according to the manufacturer's guidelines by combining Solution A and Solution B in equal parts to generate the exposure reagent. This reagent was evenly applied to the PVDF membrane to ensure complete coverage. Protein bands were detected using a gel imaging system (Thermo Fisher Scientific, USA), and the results were documented for further evaluation.

### Co‐Culture of MM Cells and NK Cells

RPMI‐8226 or U266 cells in the logarithmic growth phase were utilized as the multiple myeloma (MM) cell model, while human NK cells or NK‐92 cells served as the effector cells. Four experimental groups were established: a Control group, an ABT‐888 treatment group (treated solely with ABT‐888), an SMI‐16a treatment group (treated solely with SMI‐16a), and a Combination group (treated with both ABT‐888 and SMI‐16a). MM cells were first exposed to the respective drugs for 48 h, followed by washing with PBS to eliminate the effects of the drugs on NK cells before co‐cultivating with NK or NK‐92 cells. NK or NK‐92 cells were co‐cultured with MM cells at a 1:2 effector‐to‐target cell ratio. Each group received the appropriate drug concentrations within the therapeutic range. The co‐culture was conducted in NK‐92 medium, supplemented with 100 U mL^−1^ of interleukin‐2 (IL‐2) to promote the growth of NK‐92/NK cells. After 48 h of co‐culture, cells were harvested for flow cytometric analysis. CD138 antibodies were employed to identify myeloma cells, while CD45 antibodies were used to identify NK and NK‐92 cells. This analysis evaluated tumor cell apoptosis and assessed the expression levels of NKG2D, Granzyme B, and Perforin on NK cells.

### In Vivo Study in NSG Mice

This study was approved by the Ethics Committee of Tianjin Medical University General Hospital (Approval Number: IRB2024‐DW‐62). Male NSG mice, aged 4–6 weeks, were obtained from Dongfang Breeding Co., Ltd. in Pizhou City (Production License No.: SCXK(Su)2022‐0005). The mice were housed in a controlled environment with a 12‐h light/dark cycle to maintain circadian rhythms, and the room temperature was kept between 18 and 20 °C with relative humidity ranging from 40% to 60%.

To induce tumor formation, RPMI‐8226 multiple myeloma cells were injected subcutaneously into the NSG mice. Each mouse received 1 × 10^7^ RPMI‐8226 cells suspended in a 1:1 mixture of pre‐chilled, sterile 1 × PBS and Matrigel, with a total injection volume of ≈150 µL. The injection was administered subcutaneously in the axillary region. After 7 days of observation, the successful establishment of the tumor model was confirmed by the palpable tumor at the injection site.

When the tumor volume reached ≈100 mm^3^, the tumor‐bearing NSG mice were randomly assigned to six treatment groups, each containing four mice. The groups were as follows: Blank Control, ABT‐888 Monotherapy, SMI‐16a Monotherapy, ABT‐888 plus SMI‐16a Combination Therapy, NK Cell Treatment, and Combination plus NK Cell Treatment. Mice in each group received their respective treatments. For ABT‐888 and SMI‐16a, subcutaneous injections were administered at doses of 10 and 20 mg kg^−1^, respectively, every other day for a total of 16 days. In the NK Cell Treatment and Combination plus NK Cell Treatment groups, human NK cells were injected via tail vein at a dose of 2 × 10^7^ cells per mouse, on days 0 and 10, alongside the drug or solvent injections.

Euthanasia was performed at the end of the experiment in compliance with IACUC guidelines, and blood and relevant tissue samples were collected for subsequent analysis.

### Statistical Methods

Experimental data were presented as means ±standard deviations (SD). One‐Way Analysis of Variance (ANOVA) was used for comparisons among multiple groups. When ANOVA indicated significant differences, further analysis was conducted using Student's t‐test. A p‐value of less than 0.05 was considered statistically significant. For the quantitative analysis of image data from immunofluorescence and Western blot assays, ImageJ software was employed to accurately measure protein expression levels and fluorescence signal intensity. Flow cytometry data were processed using FlowJo software. Unless specified otherwise, all samples were analyzed in triplicate (n = 3).

### Ethics Approval

This study was approved by Ethics Committee of Tianjin Medical University General Hospital (Approval Number: IRB2024‐DW‐62).

## Conflict of Interest

The authors declare no conflict of interest.

## Author Contributions

Z.L. and W.L. contributed equally to this work as co‐first authors. R.F. and Z.L. conceptualized the experimental design and provided guidance throughout the writing process. W.L. and X.L. conducted all experiments and drafted the main body of the manuscript. X.L.,H.L., K.D., and S.J. revised and polished the paper.

## Supporting information



Supporting Information

## Data Availability

The data that support the findings of this study are available from the corresponding author upon reasonable request.
