# Supplementary material for: Combining PIM‐2 and PARP1 Inhibitors Induces MICA Expression on Multiple Myeloma Cells to Activate NK Cells through NKG2D Binding
Source: Adv Sci (Weinh). 2025 Jun 25;12(32):e02448. doi: 10.1002/advs.202502448 (PMC12407319; doi:10.1002/advs.202502448)
Supplement: Supplementary file 1 — Supporting Information [file ADVS-12-e02448-s001.doc]

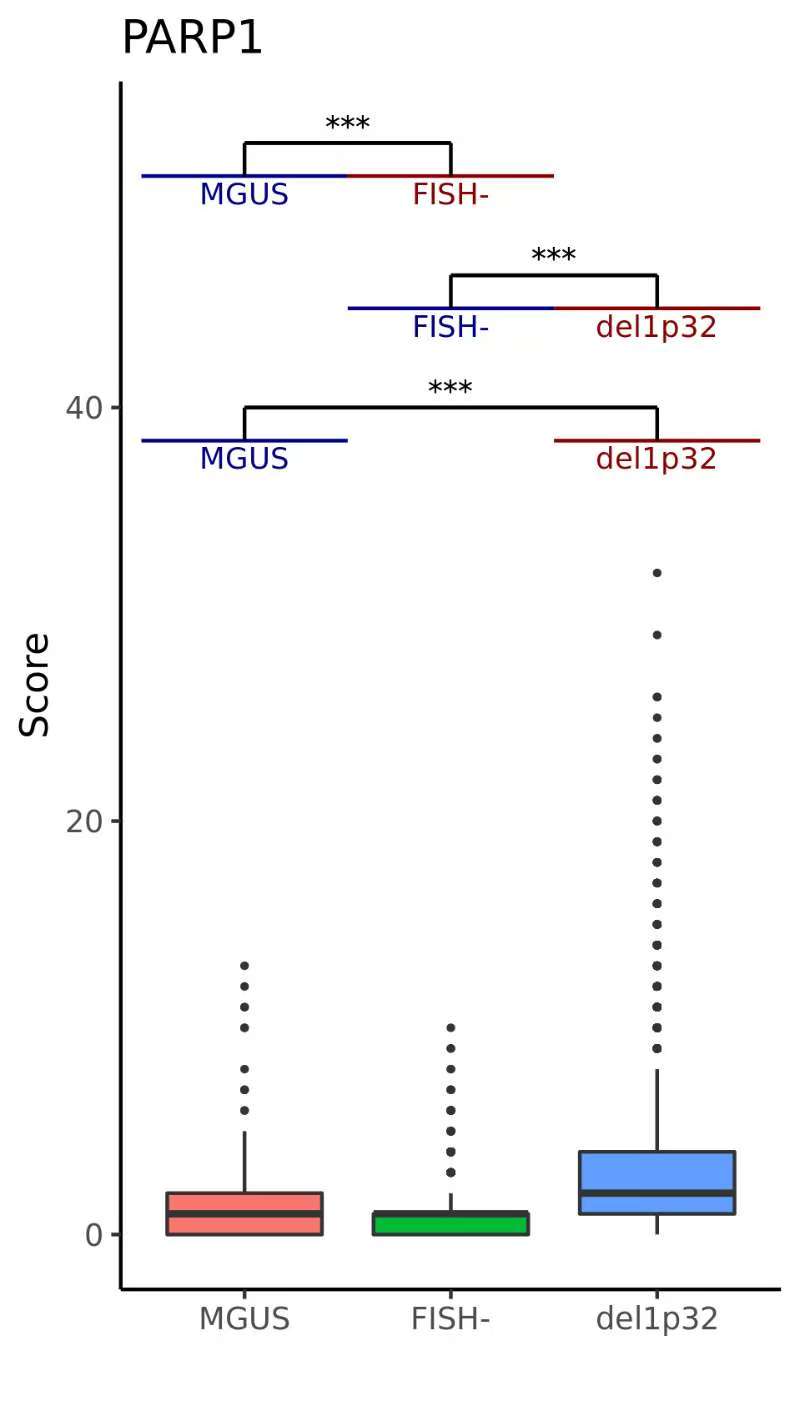


Supplementary Material Figure 1 : Single-cell sequencing results illustrate that PARP1 expression is significantly elevated in high-risk multiple myeloma (del(17p)) compared to intermediate-risk multiple myeloma (FISH negative) and monoclonal gammopathy of undetermined significance (MGUS). Note: FISH negative = intermediate-risk MM; del(17p) = high-risk MM. ***P < 0.001.


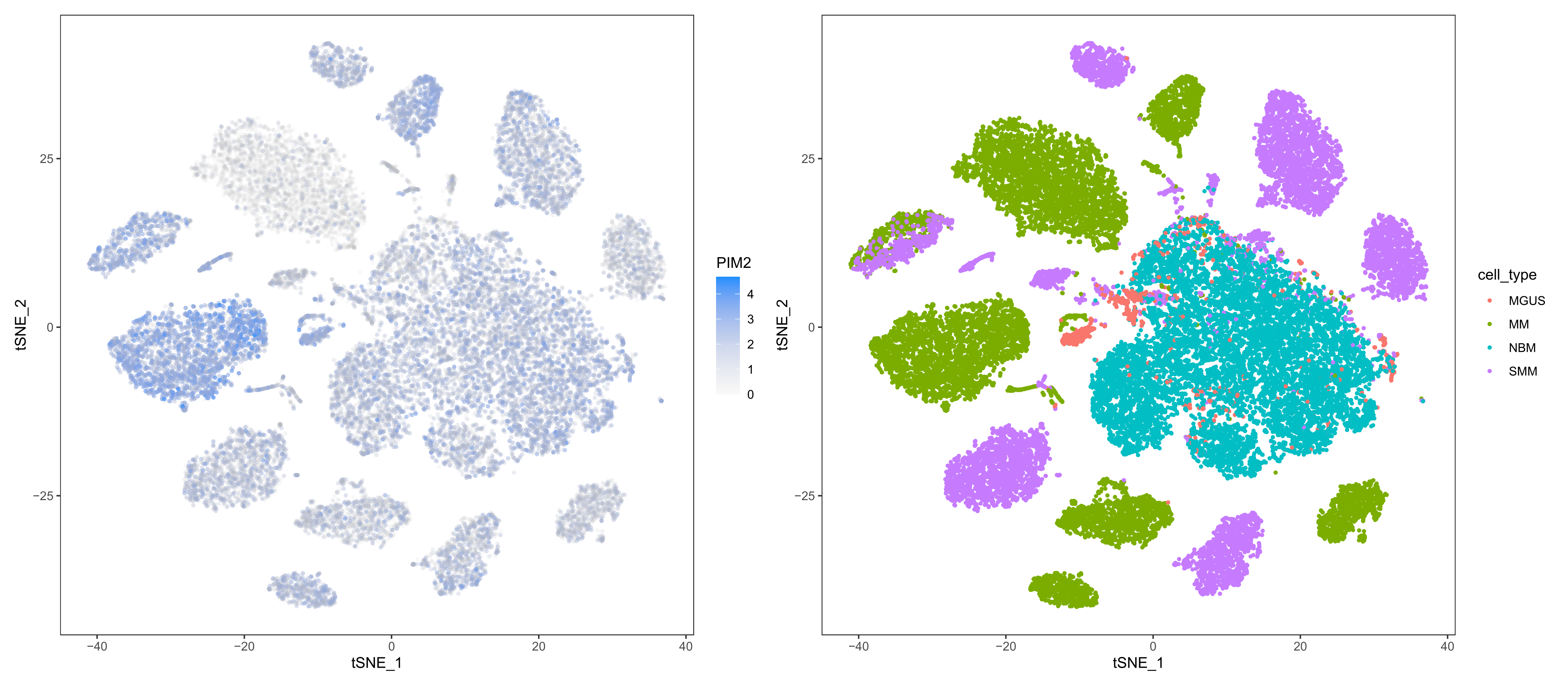


Supplementary Material Figure 2: Analysis of single-cell data from GSE193531 indicates that PIM-2 expression is markedly increased in multiple myeloma compared to normal bone marrow, MGUS, and smoldering multiple myeloma (SMM).


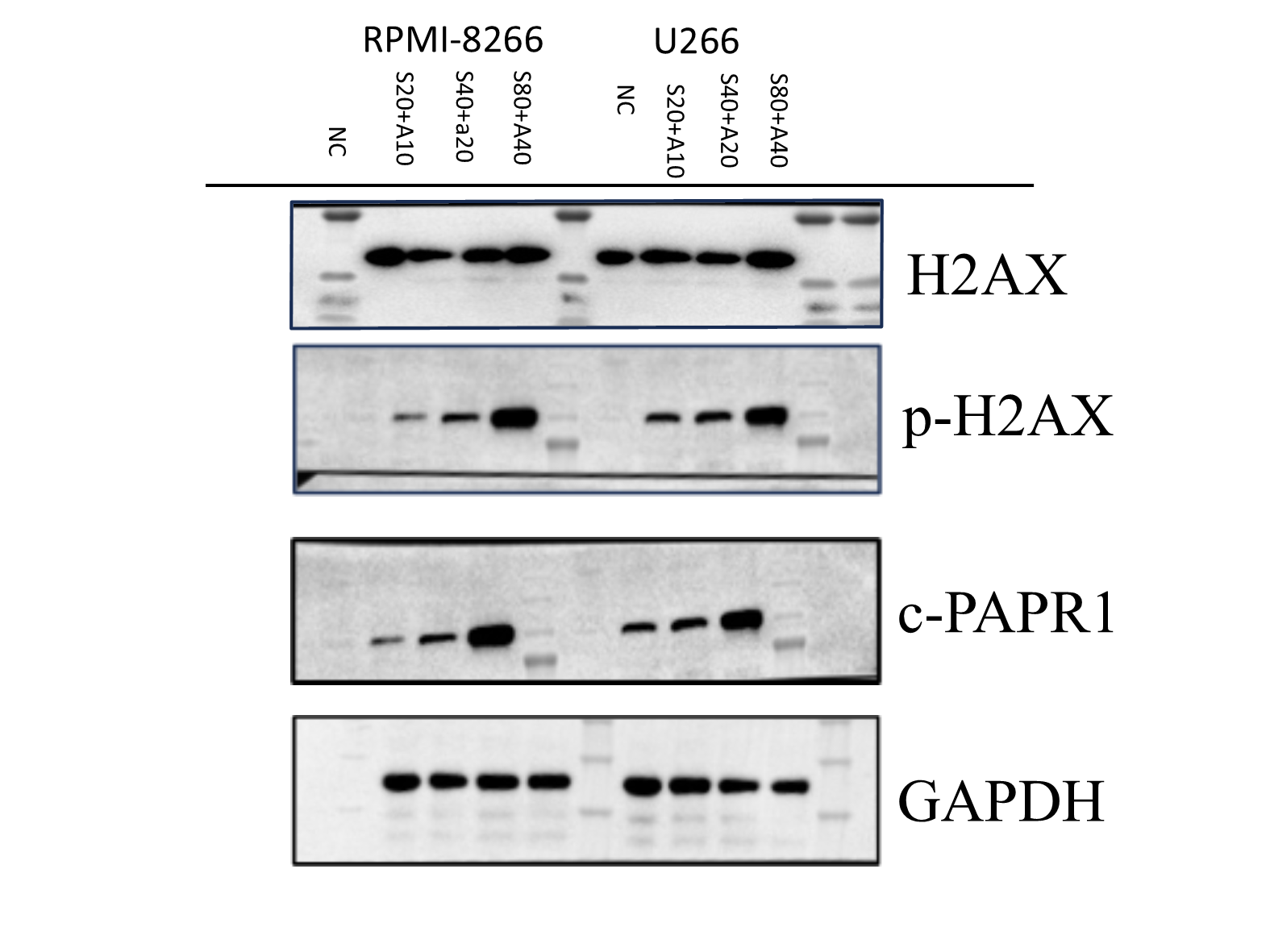


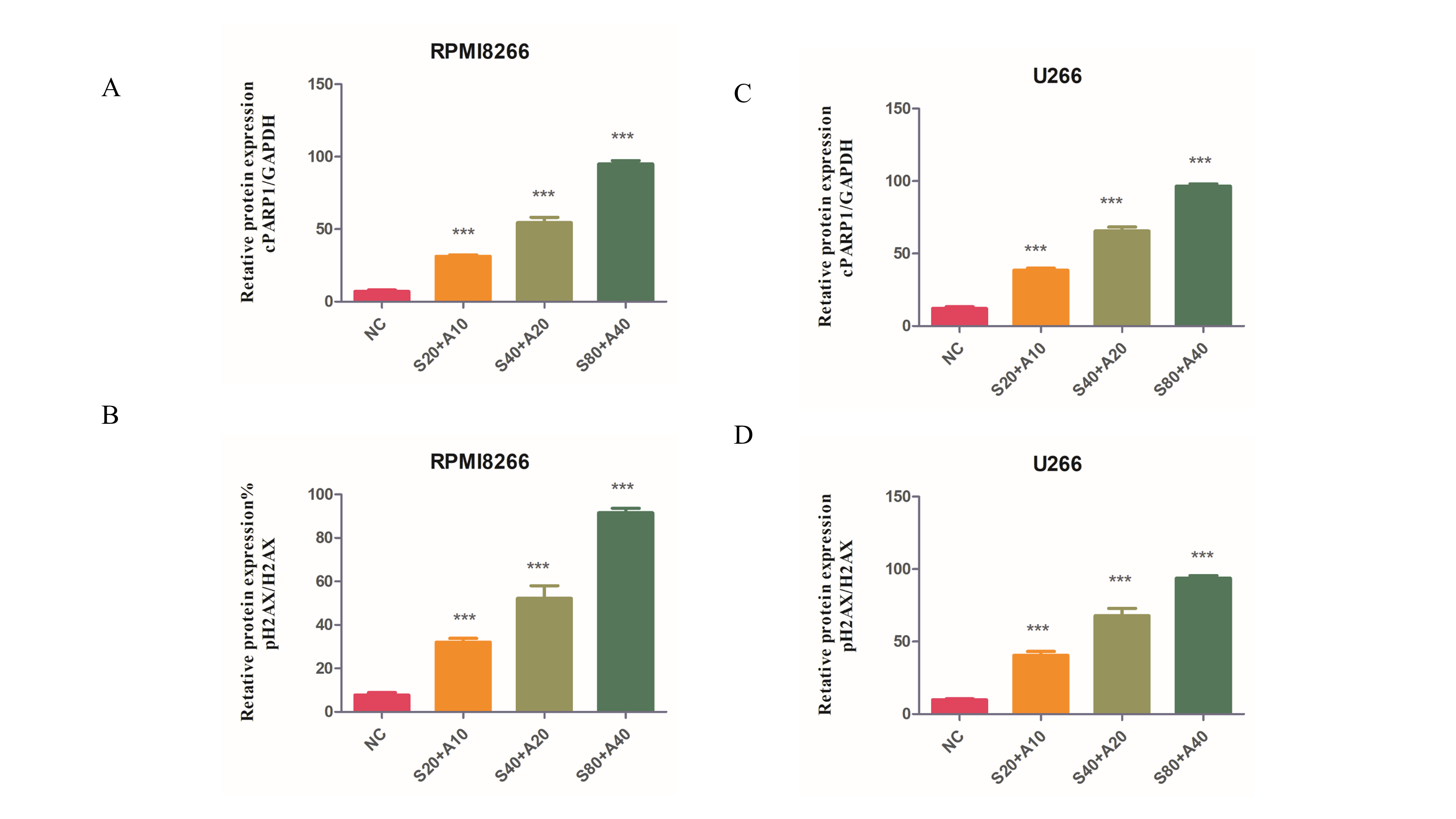


Supplementary Material Figure 3. Dose-dependent effects of combinatorial therapy on p-H2AX and PARP1 expression in MM cells.(A, B) RPMI-8226 cells; (C, D) U266 cells. Data represent mean ± SD of three independent experiments.Drug concentrations: SMI-16a (PIM2 inhibitor): 20 μM (S20), 40 μM (S40), 80 μM (S80); ABT-888 (PARP1 inhibitor): 10 μM (A10), 20 μM (A20), 40 μM (A40).Statistical significance: *P < 0.001 vs. control (NC, vehicle-treated cells) .

To deeply investigate the mechanism of dual inhibition of PIM-2 and PARP1 to promote MM cell apoptosis, we performed RNA sequencing after co-treatment of U266 and RPMI-8226 cell lines with SMI-16a and ABT888. (The subgroups were U266 group,U266-Treated group, RPMI-8226 group, RPMI-8226-Treated group) The RNA sequencing results showed that there was a significant change in the expression of genes related to DNA damage after inhibition of PIM-2 and PARP1 (Fig.4A), which were then subjected to GO pathway enrichment (Fig.4 B), and finally the enriched pathway-associated proteins were validated by Western-blot (Fig.4C). It was demonstrated that after inhibiting PIM-2 and PARP1, the phosphorylation levels of ATM and ATR increased, leading to changes in the expression of downstream related mRNAs, which not only exacerbated the DNA damage in MM cells, but also inhibited the DNA repair process at the same time, thus promoting the apoptosis level of MM cells.


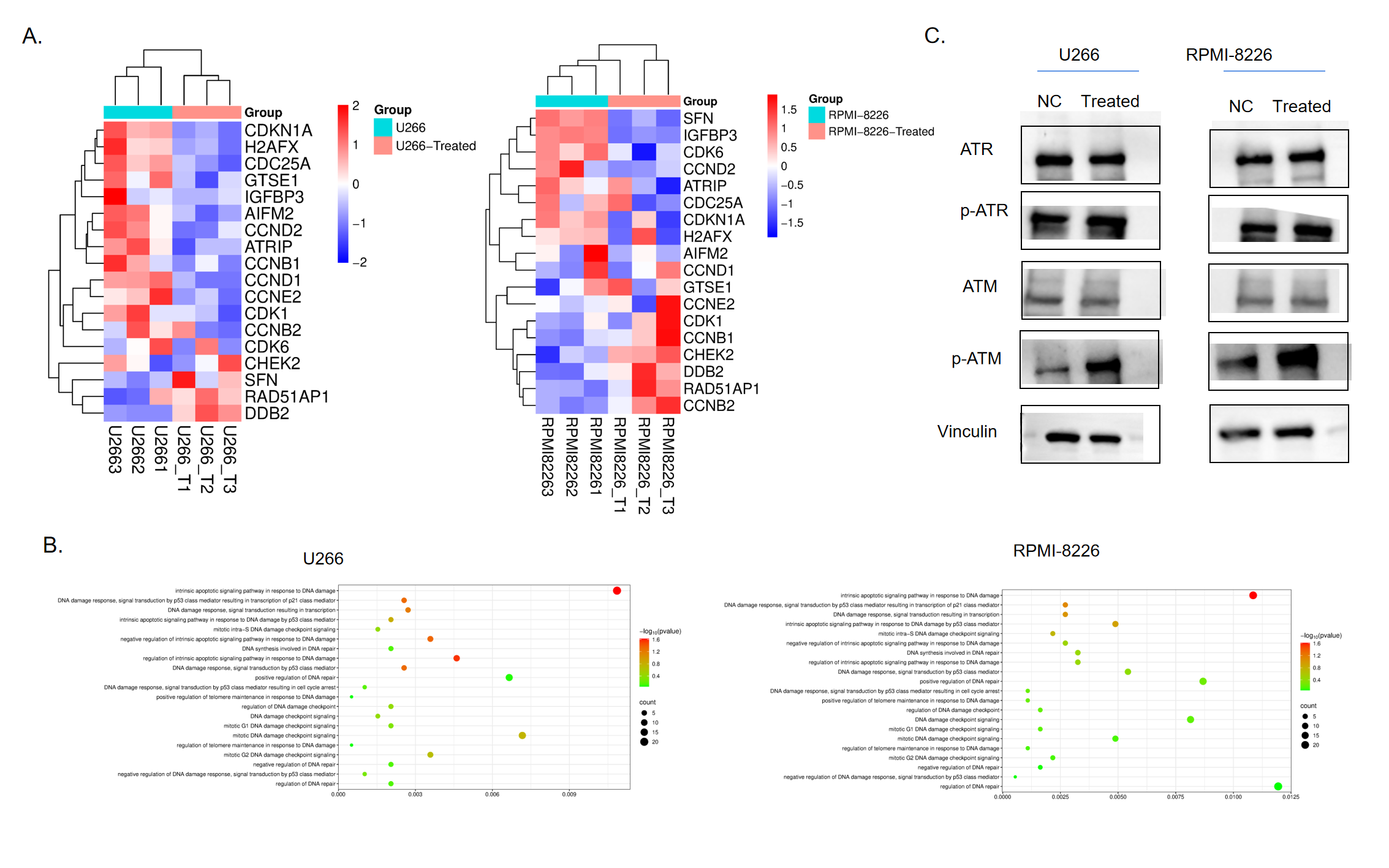


Supplementary Material Figure 4.. Probing the mechanism by which dual inhibition of PIM-2 and PARP1 promotes apoptosis in MM cells. (A) Heatmap results of RNA sequencing after treatment with U266 and RPMI-8226, respectively. (B) Relevant genes with changes after U266 and RPMI-8226 treatments were analyzed for GO pathway enrichment, showing that pathway enrichment was mainly related to DNA damage. (C) Western-blot validated RNA sequencing showed increased expression of p-ATM and p-ATR.
